# Reactive scope model and emergency life history stage provide useful tools for evaluating the stress responses of native Australian lizards living in disturbed landscapes

**DOI:** 10.1093/conphys/coab097

**Published:** 2021-12-28

**Authors:** Harsh Kirpal Pahuja, Edward Jitik Narayan

**Affiliations:** School of Agriculture and Food Sciences, Faculty of Science, University of Queensland, St Lucia, Queensland 4072, Australia

**Keywords:** survival, stress, reproduction, reactive scope model, glucocorticoids, Emergency life history stage

## Abstract

Glucocorticoids (GCs) are used as biomarkers of physiological stress response in reptiles. Fundamental stress physiology tools including the emergency life history stage (ELHS) and the reactive scope model (RSM) can be useful to determine how individual variation of stress responses shape population ecology. In this perspective, we applied the RSM and ELHS into the context of two urban-dwelling small native Australian reptile species to compare the stress-response patterns in short- and long-breeding lizards. Firstly, by drawing inferences from the ELHS, we presented hypothetical scenarios using sample GC data for a short-breeding species (e.g. common blue-tongue lizard). We showed that activation of the physiological stress response would be non-adaptive due to the consequences of stress on reproduction. Therefore, blue-tongue lizards may become exposed to acute and chronic environmental stressors (e.g. human disturbance and habitat clearance) during the breeding season as they prefer not to activate their hypothalamo-pituitary interrenal (HPI) axis in support of their short-breeding season. On the contrary, long-breeding lizards (e.g. bearded dragons), which have multiple breeding opportunities and are accustomed to living around humans and altered urban environments, tended to operate above the critical adaptive value of the ELHS during their breeding period. This suggests that any future changes to the dynamics of habitat availability and breeding opportunities may favour the dragon differently over the blue-tongue lizard. To further capture the dynamics of stress responses along spatial and temporal scales, we suggested that researchers should collect field data (e.g. blood plasma or faecal GCs) and then use the ELHS and RSM to understand how the environment is shaping the animal’s stress physiology. The application of field stress monitoring and data visualization using the ELHS and RSM could guide environmental monitoring and conservation programs of native wildlife species.

## Teaser

Stress impacts on native wildlife because of added pressure from human-induced environmental change. The intrinsic variation in physiological stress responses [e.g. glucocorticoids (GCs)] can be evaluated using the emergency life history stage (ELHS), which has an adaptive value. The temporal and spatial variation in stress responses can also be evaluated using the reactive scope model (RSM). Using the blue-tongue lizard and the Eastern bearded dragon as examples we showed how short- and long-season breeding patterns impacted the adaptive value of the ELHS. Further, with the availability of field endocrinology data (e.g. plasma or faecal GCs), researchers can scope their study species’ stress responses using the RSM to guide conservation and management programs.

## Introduction

Australia’s ‘megadiverse’ landscape has unique wildlife biodiversity, with 87% of mammal species, 93% of reptiles, 94% of frogs and 45% of bird species only found in Australia (Chapman, 2008). Australia’s unique biodiversity is declining rapidly, with more than 1700 species known to be threatened and at risk of extinction. Human-induced environmental change is one of the biggest environmental threats facing Australian wildlife (Brearley *et al.*, 2009). Further research is needed on causal relationships between environmental factors and host stress physiology (Narayan, 2017).

Recent data from wildlife hospitals and rescue centres in Australia indicate that, over the years, more and more reptiles are being rescued in urban areas due to anthropogenic-induced environmental factors such as habitat disturbance and vehicle collision (Scheelings, 2015; Taylor-Brown *et al.*, 2019). In this perspective article, using hypothetical data, we provide new insights into the potential applications of fundamental stress physiology theory and models in native species conservation and management programs. We will use examples of two local native Australian reptilian species (lizards) to show how stress physiology data when integrated with fundamental concepts, the emergency life history stage (ELHS) and reactive scope model (RSM) could increase our knowledge of species response to extreme environmental change.

## Stress, Stressors and Stress Response

Stress is an inevitable part of an animal’s life because of the rapidly changing dynamics of time and space of the environment that it inhabits (Monaghan and Haussmann, 2015). Many of the extrinsic factors that an animal comes across throughout its life history, such as circadian cycles, passing of the seasons, tidal cycles and so forth, are predictable due to intrinsic biological and evolutionary mechanisms such as biological clocks, biological (circadian and seasonal) rhythms, specific movement patterns, activity patterns, etc. These mechanisms allow an animal to cope with predictable changes or, in other words, to survive (Guindre-Parker, 2020; Monaghan and Haussmann, 2015). The physiological stress response is also an important biological mechanism that enables animals to cope with natural and anthropogenic-induced environmental changes (Wingfield, 2013a).

## The Concept of Stress and Stressors

A distinct definition of stress cannot be found in the literature and this ambiguity is primarily due to two reasons: firstly, as explained by Berkvens (2012), the concept of stress is complex with synergistic interactions among several intrinsic (animal related) and extrinsic (environmental related) factors that are difficult to ascertain, and hence the definition of stress is vague. Secondly, ‘stress’ is employed to characterize a number of related but distinctly different phenomena (Tokarz and Summers, 2011). This view is supported by Guillette *et al.* (1995) and Romero (2004), which explains that stress is used to refer to (i) external pernicious stimuli-causing stress, (ii) state of strain produced by the organism due to these stimuli and (iii) overexertion of stress responses causing bodily deteriorations. Although the aforementioned phenomena are closely related to each other, using the same term for defining different phenomena is the reason why ‘stress’ lacks a clear definition (Denardo, 2006).

As described by Selye (1950), this is an individual’s physiological (and behavioural) response to a noxious stimulus, and thus a stimulus that might induce a response from one individual might not induce a response from another individual of the same and/or different species (see Cockrem, 2013 for an extensive review on the importance of accounting for individual variation in stress studies). For example, when a group of eastern fence lizards (*Sceloporus undulatus*) were exposed to invasive ants, the stress response within experimental population of lizards varied significantly (Graham *et al.*, 2012), highlighting inter-individual differences in stress response. Furthermore, a stressor that induces a stress response at a given point of time might not induce a similar response at a different point in time for the same individual (Romero *et al.*, 2009). For example, as shown by Moore and Mason (2001), red-sided garter snakes (*Thamnophis sirtalis*) elicited a stress response when manually captured during summer; however, no change in stress response was observed when captured during late spring. This intra-individual variation can thus be regarded as an example of the context-dependent nature of stress responses in animals, which is mostly associated with crucial life history stages such as breeding.

Fundamental stress physiology concepts such as the ELHS and RSM can be customized to suit a specific research program to understand the context-dependent nature of stress responses. They will also allow us to understand and predict normal variation of physiological mediators (such as GCs) over crucial life history stages (Wingfield, 1998,2013a) and explain the impact of their variation at an inter- and intra-individual level (Romero *et al.*, 2009).

For this review, stress is described as an imbalance in the homeostasis of an individual when it is exposed to any form of stressor (Selye, 1950). Consequently, a stressor is any external stimulus that has the potential to disrupt the homeostatic equilibrium of an individual (Pryce and Fuchs, 2017). When a stressor disturbs the homeostatic equilibrium, the body adopts several physiological (and behavioural) changes to counteract the impact induced by the stressor, and to re-establish homeostasis, and this is termed as a stress response (Denardo, 2006; Tokarz and Summers, 2011). An acute stress response may be necessary to ensure survival and allow adaptation to environmental change; however, chronic stress is a problem as significant modulation of the physiological stress response can threaten animal well-being by exerting deleterious effects on the individual’s biological state.

## Mechanism of Stress Response in Reptiles

When an individual is exposed to a stressor, it makes some basic physiological (and behavioural) alterations that are common regardless of the specifics of the stressor, and these changes allow an individual to maximize its chances of survival (Denardo, 2006). These fundamental alterations involve diverting mobilized energy reserves only to those systems that are vital for survival and inhibiting the energy diversion to non-essential body systems (Denardo, 2006; Norris and Carr, 2020). When an animal is exposed to any physical and/or physiological stressor (e.g. being chased by a predator), it stimulates the paraventricular nucleus of the hypothalamus to secrete a variety of adrenocorticotrophic hormone (ACTH)-releasing factors (Aguilera, 1998), of which corticotrophin-releasing hormone (CRH) (Kabelik, 2021; Silvestre, 2014; Sinervo and Miles, 2011 ; Tokarz and Summers, 2011) and arginine vasotocin (AVT) are of prime importance in reptiles. Predominantly, CRH (and to a lesser extent AVT) is carried to the anterior pituitary via the hypothalamic–pituitary portal system where they stimulate the corticotropic cells of anterior pituitary to secrete ACTH (Sinervo and Miles, 2011; Tokarz and Summers, 2011). ACTH is then carried via the systemic blood circulation to the interrenal glands where it stimulates the synthesis and secretion of GCs (Carsia and John-Alder, 2003; Sinervo and Miles, 2011).

A characteristic feature in stress response of reptiles (and birds) is the secretion of corticosterone (CORT), instead of cortisol (as is the case in fish and mammals) as the primary stress GC (Berkvens *et al.*, 2013; Scheun *et al.*, 2018). The levels of CORT in the tissues will then rise and assist in sequestering energy to those body systems that will allow the reptile to maximize its chances of survival in that situation, either by fleeing from or fighting off the predator in that situation (Jessop *et al.*, 2015; Neuman-Lee *et al.*, 2020). However, as shown in the recent study by Neuman-Lee *et al.* (2020) the relationship between CORT and energy mobilization (e.g. glucose availability to tissues) is complex and requires further experimental validation for each species. At a cellular level CORT is thought to stimulate a decrease in cellular metabolism across the body during exposure to acute stressors, therefore transiently increasing the amount of glucose in the blood for the brain’s optimal function (see review by Kuo *et al.*, 2015 for a detailed explanation of how GCs regulate glucose).

**Figure 1:**
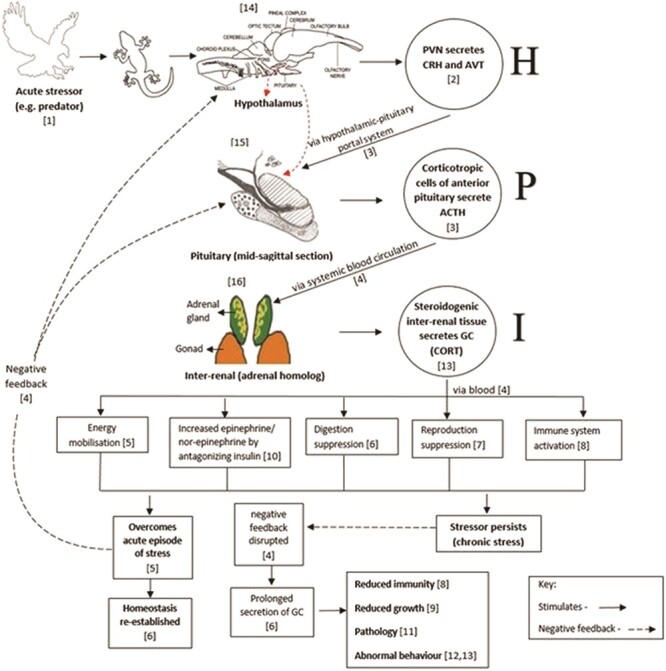
A diagram illustrating the general mechanism of physiological stress response in reptiles. Numbers in square brackets indicate references specific to adjoining comment: [1] Graham *et al.* (2012), [2] Sheriff *et al.* (2011), [3] Pecoraro *et al.* (2006), [4] Berkvens (2012), [5] Neuman-Lee *et al.* (2020), [6] Narayan (2019), [7] Moore and Jessop (2003), [8] Morici *et al.* (1997), [9] Holliday *et al.* (2009), [10] Thaker *et al.* (2010), [11] Romero *et al.* (2009), [12] Dunlap (1995), [13] Silvestre (2014), [14] Wyneken (2007), [15] Malashetty *et al.* (2009) and [16] Di Lorenzo *et al.* (2020).

On a theoretical perspective, as an example, once the reptile overcomes an acute stressor, the high level of GC is identified by the GC receptors and the GC secretion is inhibited to achieve homeostasis, by inhibiting CRH and AVT secretion at the hypothalamus level and ACTH at the pituitary level via the negative feedback loop (Berkvens, 2012). The aforementioned situation is a classic example of an acute stress response; however, it is important to note that if the reptile is exposed to a stressor for a prolonged period of time (either in terms of duration and/or frequency), it will result in prolonged and/or frequent activation of the HPA axis, which will disrupt the normal functioning of the negative feedback loop mechanism and will eventually have negative impacts on its different body systems (Narayan, 2019). The mechanism of neuroendocrine stress response and its effect on body systems in reptiles is illustrated in Fig. 1. Although not discussed in this paper, it is important to note that the sympatho–adreno–medullary system and neurohormones such as catecholamines are also important aspects of acute stress responses in reptiles (Di Lorenzo *et al.*, 2020).

## GC Plasticity and Role of Stress Physiology Models

GC plasticity refers to the varying levels of GCs expressed by a single individual across different contexts such as different environmental perturbations (Guindre-Parker, 2020). An extensive review of the literature by Injaian *et al.* (2020) evaluated baseline and stress-induced CORT levels in reptiles (measured using blood plasma samples), however, could not establish a clear association between CORT and environmental stress. Cockrem (2013) also suggests that there is a significant inter- and intra-individual difference between stress responses evoked by individuals. This variation of individual responses may be influenced by several factors such as sex, pre- and post-natal life experiences, maternal influence, position in the social hierarchy, time of the year and so on (Cockrem, 2013).

Individual variation of stress response is one of the several types of GC plasticity described by Guindre-Parker (2020) and is dictated by baseline and stress-induced GC levels. GC plasticity implies that since individuals not only portray differences in baseline and acute levels of GCs, but also in magnitude of the stress response elicited by them, it is extremely important to account for individual variation when reporting results (Guindre-Parker, 2020). For example, at a population level, urban-wild reptiles might not elicit as strong of a stress response when exposed to humans (as a stressor) as compared to their wild counterparts, since the urban-wild reptiles might be acclimatized to human presence. However, within those populations different individuals might elicit varying degrees of stress response whereas some individuals might not elicit a stress response at all, depending upon aforementioned animal-related factors as well as the context of environmental perturbation (Comendant *et al.*, 2003; Knapp and Moore, 1997; Moore *et al.*, 1991).

Stress physiology models (explained in detail further) tend to categorize individuals based on context-dependent characteristics that can be useful for accounting individual variation of stress response. In their original paper, Romero *et al.* (2009) categorizes organisms based on different characteristics such as social status (dominant versus sub-ordinate), maternal care (handled versus unhandled) and so on. Similarly, several other studies also categorize organisms based on relative extremes (Crespi *et al.*, 2013; Howell and Sanchez, 2011) inferring that although stress levels vary along a continuum, only extreme levels of stress (i.e. either too little or too much) are responsible for deleterious consequences. In our view, the major advantage of complementing stress quantification studies with theoretical models is that it will allow researchers to predict stress responses based on context-dependent categorization and back them up using empirical evidence (e.g. ACTH challenge test). Within a certain population, animal-related categorization—such as male versus female, adult versus juvenile, dominant versus sub-ordinate and so on—may all produce different results and thus categorizing the population based on the context of the study and using stress physiology models to predict and/or justify the results might prove of extreme importance to standardize the methods used to analyse stress and related phenomena.

## Overview of ELHS and RSM

### Emergency life history stage

**Figure 2:**
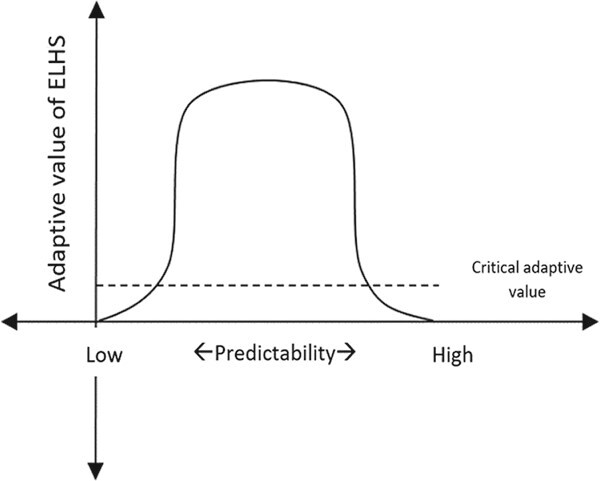
Expression of ELHS in response to predictability of perturbations, illustrating that, at extremely low and high levels of predictability, the adaptive value of ELHS drops below the ‘critical adaptive value’, as a result of which the ELHS will not be activated at these extremes. The central crest of the graph illustrates that the adaptive value of ELHS is above the ‘critical adaptive value’, as a result of which the ELHS will be activated (adapted from Wingfield, 2013a).

GCs secreted by the activity of the HPA axis play a fundamental role in modulating the transition between life history stages such as hatching, metamorphosis, sexual maturity, reproduction and so on (Crespi *et al.*, 2013). As explained by Wingfield *et al.* (1998) these ‘normal’ life history transitions are predictable and are thus mediated by intrinsic biological and evolutionary mechanisms such as circadian clock, seasonal cycles, etc. However, when an animal comes across an unpredictable situation (termed as ‘perturbation factors’) (see Wingfield *et al.*, 2011a for direct/indirect labile perturbation factors and permanent perturbation factors), the normal life history stage is disrupted and an ELHS is triggered. The ELHS involves a suite of behavioural and physiological changes that allow the individual to cope with that situation, and once the perturbation has passed, the individual returns to the normal life history stage (Wingfield *et al.*, 1998). The decision of whether or not to activate the ELHS is dependent upon the adaptive value of the ELHS, i.e. organisms are capable of eliciting a stress response or activating the ELHS only during an optimal predictable array of environmental conditions and are incapable of doing so when the environment is extremely predictable (highly stable environment) or not predictable at all (highly dynamic environment) (Wingfield *et al.*, 2011b). During these extremes, the adaptive value of eliciting a stress response or entering the ELHS is below the critical adaptive value and is thus insignificant for an animal (see Wingfield *et al.*, 2011b) (Fig. 2). This adaptive value of ELHS varies depending on different life history strategies of individuals/species/populations (Boonstra, 2013; Crespi *et al.*, 2013), and will be discussed in detail further.

**Figure 3:**
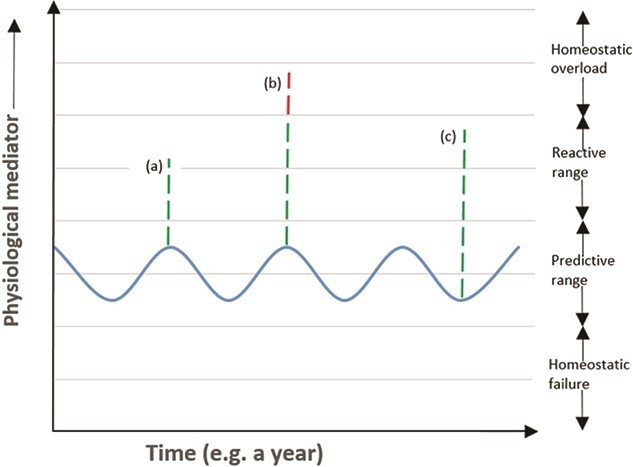
Demonstration of the RSM adapted from Romero *et al.* (2009). Descriptors (**a**) represents the predictive homeostasis, (**b**) is the reactive homeostasis range and (**c**) is the homeostatic overload have been discussed in the RSM section in-text.”””

### Reactive scope model

The RSM proposed by Romero *et al.* (2009) assists in integrating the activity of physiological mediators with unpredictable environmental changes and different life history traits, to predict the response to and impact of stressors on animals. As per the original model, the concentration of the physiological mediator fluctuates in four general ranges: Predictive Homeostasis, Reactive Homeostasis, Homeostatic Failure and Homeostatic Overload Figure (Fig. 3). The Predictive and Reactive Homeostasis range, together form the Normal Reactive Scope and fluctuation of physiological mediator beyond the Normal Reactive Scope (Romero *et al.*, 2009). The Predictive range constitutes the predictable variation of physiological mediator (i.e. circadian and seasonal rhythms, etc.); however, when an animal comes across any noxious situation (i.e. stressor), the concentration of the physiological mediator (e.g. CORT in context of this review) increases (from predictive range to reactive range, i.e. stress response) to help cope with the stressor (Fig. 3). If the animal comes across a stressor of high magnitude, the concentration of CORT will increase beyond the upper threshold of the reactive range (surpassing the Normal Reactive Scope) (Fig. 3). Long-term exposure to stressors could result in pathology and eventually death if animals are often required to maintain the upper threshold of the reactive range. Similarly, if CORT cannot be maintained above the threshold between Homeostatic Failure and Predictive Homeostasis range, this will result in Homeostatic Failure and rapid death since the physiological mediator can no longer carry out essential physiological processes (Romero *et al.*, 2009). The negative consequences of entering the homeostatic overload and/or homeostatic failure range have been summarized by Romero *et al.* (2009) in their original paper. An important caveat to note is that two stressors of equal magnitude may or may not cause CORT levels to enter homeostatic overload range depending on various intrinsic and/or extrinsic factors (e.g. life history stage) influencing the ‘normal’ variation of CORT (Romero *et al.*, 2009).

**Figure 4:**
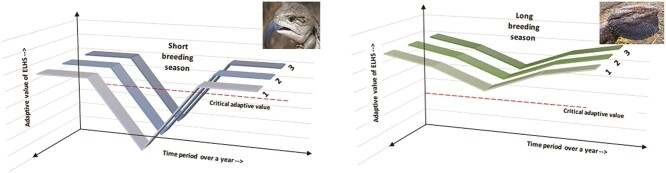
Left-hand panel: Expression of ELHS in *T. scincoides* as an example of an animal with a short-breeding season, illustrating a precipitous drop in the adaptive value during the breeding season to avoid the onset of ELHS. Right-hand panel: Expression of ELHS in *P. barbata* as an example of an animal with a long-breeding season, illustrating a slight dent in the adaptive value during the breeding season to modulate the expression of ELHS (adapted from Wingfield, 2013a). (Note: Multiple grey tracks are illustrated to indicate repeatability over several seasons/years as indicated by numbers 1–3).

### Implication of ELHS and RSM to understand coping mechanisms in reptiles

The ELHS model can be used to predict whether or not a reptile (or any animal) would initiate a stress response (i.e. transition into an ELHS) when exposed to a stressor. This prediction is based on the adaptive value of the ELHS, which is dependent on different life history strategies that have evolved over time (Boonstra, 2013; Crespi *et al.*, 2013). Breeding season is vital to study since it dictates an important life history stage in every animal’s life, i.e. reproduction/mating (Wingfield *et al.*, 1998). Consider, for example, animals with short and long breeding seasons. In case of seasonal animals with a short breeding season, for example, common blue-tongue lizard (*Tiliqua scincoides*) whose breeding season is only during spring in Australia, there do not exist multiple opportunities to mate and raise the offspring. Since the rewards of raising the offspring prevail the benefits of activating the ELHS, the adaptive value of ELHS plummets during the breeding season (Fig. 4) and, consequently, the hypothalamo-pituitary interrenal (HPI) axis response to stressors is inhibited and the ELHS is silenced (Wingfield *et al.*, 2011a). As explained by Kokko and Ranta (1996), it is only beneficial to delay reproduction if the number of future offspring produced will be greater than the current bout of reproduction. For a species like the blue-tongue lizard, this is highly unlikely, given the short seasonal breeding period and high risk of mortality associated with urban landscapes (Koenig *et al.*, 2002) and consumption of toxic cane toads (Price-Rees *et al.*, 2010). Although we do not yet have primary data on whether common blue-tongue lizards attenuate their stress responses during the breeding season, previous research on other viviparous species (e.g. *Lacerta vivipara*) had found no raise in blood CORT levels after 8 minutes of continuous restraint and blood sampling (Dauphin-Villemant and Xavier, 1987). Earlier work also showed that blue-tongue lizards actually did not generate a CORT stress response to 10 minutes of handling and restraint (Kreger and Mench, 1993). This perspective certainly opens an avenue for further line of research on the physiological coping mechanisms of blue-tongue lizards during the breeding season, especially in urban landscapes where the incidences of human-induced stressors (e.g. vehicle collisions) is very high (Scheelings, 2015; Taylor-Brown *et al.*, 2019).

On the other hand, in case of a non-seasonal animal, for example, eastern bearded dragon (*Pogona barbata*) with a breeding season from spring to summer in Australia (Hutchinson, 2018), it may have multiple opportunities to mate successfully, and therefore it might not be necessary to completely silence its ELHS. Although the adaptive value declines slightly, it never falls below the critical adaptive value (Fig. 4) and, consequently, adrenal (−interrenal) response and activation of ELHS is conserved to combat severe perturbations (Wingfield, 2013b). Similar speculations based on different life history strategies have been made by Crespi *et al.* (2013) for two hypothetical populations where population A (common blue-tongue lizard in this case) chooses reproduction over survival and population B (bearded dragon in this case) chooses survival over reproduction (i.e. future reproductive opportunity over current reproductive success). Boonstra (2013) explains that different life history strategies have evolved over time to benefit the fitness and survival of individuals/species/populations.

With the availability of the RSM (Romero *et al.*, 2009), researchers then have access to another powerful tool to evaluate the integration of the HPA-axis activity with unpredictable environmental changes and different life history traits, to predict the response to and impact of stressors on animals. By applying the RSM in Galapagos marine iguanas (*Amblyrhynchus cristatus*), Romero (2012) explains that delayed termination of stress response results in prolonged activation of ELHS and eventually causes mortality in those populations. This delayed termination of the physiological stress response results in progressive reduction of the normal reactive scope (Fig. 3) making these populations more vulnerable to any additional stressors. It can be inferred that in order to maximize the chances of their survival, these iguanas should either avoid any further stressors, or avoid eliciting a stress response when exposed to a stressor. The latter would be possible if the adaptive value of the ELHS was below the critical adaptive value, which would allow the animal to inactivate the transition into ELHS (Fig. 2).

Similarly, we apply the RSM to the previous example of short versus long breeding season lizards and predict their response to an acute stressor. Since common blue-tongue lizards have critically low adaptive value of transition into ELHS during the breeding season (Fig. 4), the transition to ELHS is silenced to facilitate the current bout of reproduction (reproduction wins over immediate survival). On the other hand, if the blue-tongue lizard does not silence the ELHS when exposed to an acute stressor during breeding period, this could make it vulnerable to enter into homeostatic overload (referring to the RSM) (Figs 3 and 4). This explanation is in line with the notion that the circulatory levels of physiological mediator (e.g. CORT) might already be within homeostatic range to facilitate the energetic demands of reproduction (Wingfield, 2013a; Wingfield and Sapolsky, 2003).

As explained by Wingfield (2013a), transitioning the HPA-axis response into the ELHS increases the probability of short-term survival of an animal to combat the stressor, through elevation of CORT levels (and other physiological and behavioural mechanisms); however, the probability of reproduction is decreased since majority of energy reserves will be diverted away in overcoming the stressor. Based on this, we predict that the bearded dragon could still afford to elicit a stress response to deal with the current stressor (immediate survival wins over reproduction), hence they would elicit a stress response to overcome the stressor because of two main reasons: (i) it can have several opportunities to mate in the future and (ii) because it is at low risk of entering the homeostatic overload range (Fig. 3). It is, however, important to note that earlier research showed that during breeding season, the bearded dragon also tends to modulate its acute stress responses to physical stressors (e.g. capture). It will be worthwhile to carryout longitudinal monitoring (e.g. within versus outside breeding season) of the stress responses of the bearded dragon to better understand how the species operates under the fundamental theories of the ELHS and RSM.

## Conclusions

The ELHS provides a powerful tool for researchers to determine how the study species utilizes its stress endocrine system during exposure to stressors. This can be evaluated by exploring the adaptive value of the ELHS and determining where the stress responses of the study species sits along the predictive range and between the lower and upper levels of the adaptive and critical adaptive values (see Figs 2 and 4). With availability of field endocrinology data, researchers will also be able to apply the RSM to make long-term assessments of the physiological response and adaptation of their study species across spatial and temporal scales (Fig. 3). We believe that there is a definite value in using these fundamental approaches to study the eco-physiology of native small reptiles in Australia such as the blue-tongue lizard and the bearded dragon and this data could be useful for conservation biologists and urban planners as well. For example, with the identification of the adaptive state of the stress response of the study species using the ELHS and by knowing the pattern of temporal and spatial variation in the stress response (using the RSM), researchers will know exactly whether their species is facing some underlying physiological problems (e.g. lizard species requiring to active the stress response to high levels during the breeding season due to increased severity of environmental stress). We certainly require this type of integrated approach and field endocrinology data to progress our knowledge on the stress physiology of native wildlife and also provide empirical evidence to show how species are responding to and are being impacted by extreme environmental change.

## References

[ref1] Aguilera G (1998) Corticotropin releasing hormone, receptor regulation and the stress-response. Trends Endocrinol Metab 9: 329–336. 18406298 10.1016/s1043-2760(98)00079-4

[ref2] Berkvens CN (2012) Keratin glucocorticoid analysis by enzyme immunoassay in mammals, birds and reptiles. Thesis. University of Guelph.

[ref3] Berkvens CN, Hyatt C, Gilman C, Pearl DL, Barker IK, Mastromonaco GF (2013) Validation of a shed skin corticosterone enzyme immunoassay in the African house snake (*Lamprophis fuliginosus*) and its evaluation in the eastern massasauga rattlesnake (*Sistrurus catenatus catenatus*). Gen Comp Endocrinol 194: 1–9. 23994033 10.1016/j.ygcen.2013.08.011

[ref4] Boonstra R (2013) Reality as the leading cause of stress: rethinking the impact of chronic stress in nature. Funct Ecol 27: 11–23.

[ref5] Brearley G, Rhodes J, Bradley A, Baxter G, Seabrook L, Lunney D, Liu Y, McAlpine C (2013) Wildlife disease prevalence in human-modified landscapes. Biol Rev Camb Philos Soc 88: 427–442. 23279314 10.1111/brv.12009

[ref6] Carsia RV, John-Alder H (2003) Seasonal alterations in adrenocortical cell function associated with stress-responsiveness and sex in the eastern fence lizard (*Sceloporus undulatus*). Horm Behav 43: 408–420. 12695115 10.1016/s0018-506x(03)00013-8

[ref7] Chapman, A.D. (2008) Numbers of living species in Australia and the world. Report for the Australian Biological Resources Study. Department of the Environment, Water, Heritage and the Arts, Australian Government, Canberra, Australia.

[ref8] Cockrem JF (2013) Individual variation in glucocorticoid stress-responses in animals. Gen Comp Endocrinol 181: 45–58. 23298571 10.1016/j.ygcen.2012.11.025

[ref9] Comendant T, Sinervo B, Svensson EI, Wingfield J (2003) Social competition, corticosterone and survival in female lizard morphs. J Evol Biol 16: 948–955. 14635910 10.1046/j.1420-9101.2003.00598.x

[ref10] Crespi EJ, Williams TD, Jessop TS, Delehanty B (2013) Life history and the ecology of stress: how do glucocorticoid hormones influence life-history variation in animals? Funct Ecol 27: 93–106.

[ref11] Dauphin-Villemant C, Xavier F (1987) Nychthemeral variations of plasma corticosteroids in captive female Lacerta vivipara Jacquin: influence of stress and reproductive state. Gen Comp Endocrinol 67: 292–302. 3666407 10.1016/0016-6480(87)90183-3

[ref12] Denardo D (2006) Stress in captive reptiles. In Reptile Medicine and Surgery. Elsevier, Philadelphia, Pennsylvania, United States, pp. 119–123

[ref13] Di Lorenzo M, Barra T, Rosati L, Valiante S, Capaldo A, De Falco M, Laforgia V (2020) Adrenal gland response to endocrine disrupting chemicals in fishes, amphibians and reptiles: a comparative overview. Gen Comp Endocrinol 297: 113550. 32679158 10.1016/j.ygcen.2020.113550

[ref14] Dunlap KD (1995) Hormonal and behavioral responses to food and water deprivation in a lizard (*Sceloporus occidentalis*): implications for assessing stress in a natural population. J Herpetol 29: 345–351.

[ref15] Graham SP, Freidenfelds NA, McCormick GL, Langkilde T (2012) The impacts of invaders: basal and acute stress glucocorticoid profiles and immune function in native lizards threatened by invasive ants. Gen Comp Endocrinol 176: 400–408. 22226759 10.1016/j.ygcen.2011.12.027

[ref16] Guillette LJ, Cree A, Rooney AA (1995) Biology of stress: interactions with reproduction, immunology and intermediary metabolism. In Health and Welfare of Captive Reptiles. Springer, Berlin/Heidelberg, Germany, pp. 32–81

[ref17] Guindre-Parker S (2020) Individual variation in glucocorticoid plasticity: considerations and future directions. Integr Comp Biol 60: 79–88. 32101288 10.1093/icb/icaa003

[ref18] Holliday DK, Elskus AA, Roosenburg WM (2009) Impacts of multiple stressors on growth and metabolic rate of malaclemys terrapin. Environ Toxicol Chem 28: 338–345. 18788897 10.1897/08-145.1

[ref19] Howell BR, Sanchez MM (2011) Understanding behavioral effects of early life stress using the reactive scope and allostatic load models. Dev Psychopathol 23: 1001. 22018078 10.1017/S0954579411000460 PMC4593415

[ref20] Hutchinson M (2018) *Pogona barbata*. The IUCN Red List of Threatened Species (T170419A83493237).

[ref21] Injaian AS, Francis CD, Ouyang JQ, Dominoni DM, Donald JW, Fuxjager MJ, Goymann W, Hau M, Husak JF, Johnson MA et al. (2020) Baseline and stress-induced corticosterone levels across birds and reptiles do not reflect urbanization levels. Conserv Phys Ther 8: coz110. 10.1093/conphys/coz110 PMC6978728 31993201

[ref22] Jessop TS, Anson JR, Narayan E, Lockwood T (2015) An introduced competitor elevates corticosterone responses of a native lizard (*Varanus varius*). Physiol Biochem Zool 88: 237–245. 25860823 10.1086/680689

[ref23] Kabelik D (2021) Corticotropin-releasing factor distribution in the brain of the brown anole lizard. bioRxiv.

[ref24] Knapp R, Moore MC (1997) Male morphs in tree lizards have different testosterone responses to elevated levels of corticosterone. Gen Comp Endocrinol 107: 273–279. 9245535 10.1006/gcen.1997.6923

[ref25] Koenig J, Shine R, Shea G (2002) The dangers of life in the city: patterns of activity, injury and mortality in suburban lizards (*Tiliqua scincoides*). J Herpetol 36: 62–68.

[ref26] Kokko H, Ranta E (1996) Evolutionary optimality of delayed breeding in voles. Oikos 77: 173–175.

[ref27] Kreger MD, Mench JA (1993) Physiological and behavioral effects of handling and restraint in the ball python (*Python regius*) and the blue-tongued skink (*Tiliqua scincoides*). Appl Anim Behav Sci 38: 323–336.

[ref28] Kuo T, McQueen A, Chen TC, Wang JC (2015) Regulation of glucose homeostasis by glucocorticoids. Glucocorticoid Signaling, 872: 99–126 10.1007/978-1-4939-2895-8_5 PMC6185996 26215992

[ref29] Lendvai AZ, Ouyang JQ, Schoenle LA, Fasanello V, Haussmann MF, Bonier F, Moore IT (2014) Experimental food restriction reveals individual differences in corticosterone reaction norms with no oxidative costs. PLoS One 9: e110564. 25386675 10.1371/journal.pone.0110564 PMC4227652

[ref30] Malashetty VB, Sonar A, Patil SB (2009) Anatomy and histophysiological changes in pituitary of calotes versicolor during breeding and nonbreeding seasons. Int J Morphol 27: 1223–1234.

[ref31] Monaghan P, Haussmann MF (2015) The positive and negative consequences of stressors during early life. Early Hum Dev 91: 643–647. 26385447 10.1016/j.earlhumdev.2015.08.008 PMC4706554

[ref32] Moore IT, Jessop TS (2003) Stress, reproduction, and adrenocortical modulation in amphibians and reptiles. Horm Behav 43: 39–47. 12614633 10.1016/s0018-506x(02)00038-7

[ref33] Moore IT, Mason RT (2001) Behavioral and hormonal responses to corticosterone in the male red-sided garter snake, *Thamnophis sirtalis parietalis*. Physiol Behav 72: 669–674. 11336998 10.1016/s0031-9384(01)00413-9

[ref34] Moore MC, Thompson CW, Marler CA (1991) Reciprocal changes in corticosterone and testosterone levels following acute and chronic handling stress in the tree lizard. Gen Comp Endocr 81: 217–226. 2019396 10.1016/0016-6480(91)90006-r

[ref35] Morici LA, Elsey RM, Lance VA (1997) Effects of long-term corticosterone implants on growth and immune function in juvenile alligators, *Alligator mississippiensis*. J Exp Zool 279: 156–162. 9293640

[ref36] Narayan EJ (2017) Evaluation of physiological stress in Australian wildlife: embracing pioneering and current knowledge as a guide to future research directions. Gen Comp Endocrinol 244: 30–39. 26686317 10.1016/j.ygcen.2015.12.008

[ref37] Narayan EJ (2019) Introductory chapter: applications of stress endocrinology in wildlife conservation and livestock science. Comp Endocrinol Anim 1–8.

[ref38] Neuman-Lee LA, Hudson SB, Webb AC, French SS (2020) Investigating the relationship between corticosterone and glucose in a reptile. J Exp Biol 223: jeb203885. 10.1242/jeb.203885 31767736

[ref39] Norris DO, Carr JA (2020) Vertebrate Endocrinology. Academic Press, Cambridge, Massachusetts, United States.

[ref40] Pecoraro N, Dallman MF, Warne JP, Ginsberg AB, Laugero KD, la Fleur SE, Houshyar H, Gomez F, Bhargava A, Akana SF (2006) From malthus to motive: how the HPA axis engineers the phenotype, yoking needs to wants. Prog Neurobiol 79: 247–340. 16982128 10.1016/j.pneurobio.2006.07.004

[ref41] Price-Rees SJ, Brown GP, Shine R (2010) Predation on toxic cane toads (*Bufo marinus*) may imperil bluetongue lizards (*Tiliqua scincoides intermedia*, Scincidae) in tropical Australia. Wildl Res 37: 166–173.

[ref42] Pryce CR, Fuchs E (2017) Stressors in animals and humans-practical issues and limitations. Neurobiol Stress 6: 1. 28229103 10.1016/j.ynstr.2017.02.001 PMC5314438

[ref43] Romero LM (2004) Physiological stress in ecology: lessons from biomedical research. Trends Ecol Evol 19: 249–255. 16701264 10.1016/j.tree.2004.03.008

[ref44] Romero LM (2012) Using the reactive scope model to understand why stress physiology predicts survival during starvation in Galápagos marine iguanas. Gen Comp Endocrinol 176: 296–299. 22101208 10.1016/j.ygcen.2011.11.004

[ref45] Romero LM, Dickens MJ, Cyr NE (2009) The reactive scope model—a new model integrating homeostasis, allostasis, and stress. Horm Behav 55: 375–389. 19470371 10.1016/j.yhbeh.2008.12.009

[ref46] Scheelings TF (2015) Morbidity and mortality of reptiles admitted to the Australian wildlife health Centre, Healesville sanctuary, Australia, 2000–13. J Wildl Dis 51: 712–718. 26161722 10.7589/2014-09-230

[ref47] Scheun J, Greeff D, Ganswindt A (2018) Non-invasive monitoring of glucocorticoid metabolite concentrations in urine and faeces of the sungazer (*Smaug giganteus*). PeerJ 6: e6132. 30595985 10.7717/peerj.6132 PMC6305116

[ref48] Schoech SJ, Romero LM, Moore IT, Bonier F (2013) Constraints, concerns and considerations about the necessity of estimating free glucocorticoid concentrations for field endocrine studies. Funct Ecol 27: 1100–1106.

[ref49] Selye H (1950) Stress and the general adaptation syndrome. Br Med J 1: 1383. 15426759 10.1136/bmj.1.4667.1383 PMC2038162

[ref50] Sheriff MJ, Dantzer B, Delehanty B, Palme R, Boonstra R (2011) Measuring stress in wildlife: techniques for quantifying glucocorticoids. Oecologia 166: 869–887. 21344254 10.1007/s00442-011-1943-y

[ref51] Silvestre AM (2014) How to assess stress in reptiles. J Exot Pet Med 23: 240–243.

[ref52] Sinervo B, Miles D (2011) Hormones and behavior of reptiles. In Hormones and Reproduction of Vertebrates. Academic Press, Cambridge, Massachusetts, United States. pp. 215–246.

[ref53] Taylor-Brown A, Booth R, Gillett A, Mealy E, Ogbourne SM, Polkinghorne A, Conroy GC (2019) The impact of human activities on Australian wildlife. PLoS One 14: e0206958. 30673712 10.1371/journal.pone.0206958 PMC6344025

[ref54] Thaker M, Vanak AT, Lima SL, Hews DK (2010) Stress and aversive learning in a wild vertebrate: the role of corticosterone in mediating escape from a novel stressor. Am Nat 175: 50–60. 19922261 10.1086/648558

[ref55] Tokarz RR, Summers CH (2011) Stress and reproduction in reptiles. In Hormones and Reproduction of Vertebrates. Elsevier, Amsterdam, Netherlands, pp. 169–213

[ref56] Wingfield J, Sapolsky R (2003) Reproduction and resistance to stress: when and how. J Neuroendocrinol 15: 711–724. 12834431 10.1046/j.1365-2826.2003.01033.x

[ref57] Wingfield JC (2013a) The comparative biology of environmental stress: behavioural endocrinology and variation in ability to cope with novel, changing environments. Anim Behav 85: 1127–1133.

[ref58] Wingfield JC (2013b) Ecological processes and the ecology of stress: the impacts of abiotic environmental factors. Funct Ecol 27: 37–44.

[ref59] Wingfield JC, Kelley JP, Angelier F (2011a) What are extreme environmental conditions and how do organisms cope with them? Curr Zool 57: 363–374.

[ref60] Wingfield JC, Kelley JP, Angelier F, Chastel O, Lei F, Lynn SE, Miner B, Davis JE, Li D, Wang G (2011b) Organism–environment interactions in a changing world: a mechanistic approach. J Ornithol 152: 279–288.

[ref61] Wingfield JC, Maney DL, Breuner CW, Jacobs JD, Lynn S, Ramenofsky M, Richardson RD (1998) Ecological bases of hormone—behavior interactions: the “emergency life history stage”. Am Zool 38: 191–206.

[ref62] Wyneken J (2007) Reptilian neurology: anatomy and function. Vet Clin North Am Exot Anim Pract 10: 837–853. 17765850 10.1016/j.cvex.2007.05.004

